# Trap Exploration in Amorphous Boron-Doped ZnO Films

**DOI:** 10.3390/ma8095276

**Published:** 2015-08-31

**Authors:** Fu-Chien Chiu, Wen-Ping Chiang

**Affiliations:** Department of Electronic Engineering, Ming Chuan University, 5 De-Ming Rd., Gui-Shan, Taoyuan 33348, Taiwan; E-Mail: angie21007@hotmail.com

**Keywords:** boron doped ZnO, conduction mechanism, trap energy level, trap spacing

## Abstract

This paper addresses the trap exploration in amorphous boron-doped ZnO (ZnO:B) films using an asymmetric structure of metal-oxide-metal. In this work, the structure of Ni/ZnO:B/TaN is adopted and the ZnO:B film is deposited by RF magnetron sputtering. The as-deposited ZnO:B film is amorphous and becomes polycrystalline when annealing temperature is above 500 °C. According to the analysis of conduction mechanism in the as-deposited ZnO:B devices, Ohmic conduction is obtained at positive bias voltage because of the Ohmic contact at the TaN/ZnO:B interface. Meanwhile, hopping conduction is obtained at negative bias voltage due to the defective traps in ZnO:B in which the trap energy level is lower than the energy barrier at the Ni/ZnO:B interface. In the hopping conduction, the temperature dependence of *I-V* characteristics reveals that the higher the temperature, the lower the current. This suggests that no single-level traps, but only multiple-level traps, exist in the amorphous ZnO:B films. Accordingly, the trap energy levels (0.46–0.64 eV) and trap spacing (1.1 nm) in these multiple-level traps are extracted.

## 1. Introduction

Zinc oxide (ZnO) is an attractive material for the applications of semiconductor devices [1]. It has a direct and wide band gap in which the value is 3.37 eV at room temperature and increases to be about 3.44 eV at 4.2 K. This property makes ZnO transparent in visible light and enables optoelectronic applications in blue and ultraviolet region, such as light emitting devices, laser diodes and photosensors [2]. Additionally, the large free-exciton binding energy of 60 meV in ZnO, compared with 25 meV in GaN, is of interest to achieve excitonic stimulated emission for the realization of low-threshold lasers at or even above room temperature [3,4]. One interesting feature of ZnO is the ability of bandgap engineering by its alloying with CdO (*E_g_* = 2.3 eV) or MgO (*E_g_* ~ 7.7 eV). Namely, bandgap energy of 2.99 eV (Cd*_y_*Zn_1−*y*_O, *y* = 0.07) can be achieved by doping with Cd^2+^, while Mg^2+^ increases the bandgap energy to 3.9 eV (Mg*_x_*Zn_1−*x*_O, *x* = 0.33) [5,6,7]. ZnO can be used for phosphor applications because of a strong luminescence in the green–white region of the spectrum. The n-type conductivity of ZnO enables the applications in vacuum fluorescent displays and field emission displays [1,8].

In general, ZnO with a wurtzite structure is an unintentional n-type semiconductor because of the deviation from stoichiometry. The background free electrons basically result from the shallow donor levels related to the presence of native defects such as oxygen vacancies and/or zinc interstitials [2]. To achieve higher n-type conductivity of ZnO films, intentional n-type doping can be implemented by the substitution of Group III elements (B, Al, Ga, and In) on the Zn sites or Group VII elements (F and Cl) on the O sites [2]. After doping of Group III elements, ZnO is favorable for replacing tin oxide (SnO_2_) or indium tin oxide (ITO) as a transparent conducting electrode in liquid crystal displays or solar cell devices due to the advantages of abundant raw material, low synthetic temperature, available large single crystal, amenable wet chemical etching, simple manufacturing process, competitive optical and electrical properties, nontoxic and stable in plasma, and radiation hardness [1,9]. Note that the Group III elements used for doping ZnO to enhance conductivity are substituting Zn atoms in the host lattice. Although there are some literature studies advocating that suggestion, there is no conclusive evidence yet. The Group III elements may exist as interstitials instead of substituting the Zn atoms in the host lattice [1]. Recently, ZnO-based diluted magnetic semiconductors showed ferromagnetism in ZnO by doping with boron or a transition metal, which is promising to achieve practical Curie temperature for future spintronic devices [3,4]. In addition, transparent boron-doped ZnO (ZnO:B) films sandwiched between two tungsten electrodes showed memristive behavior, which is attractive to overcome the physical limitations of traditional Flash memory for the next generation nonvolatile memory applications [10].

Because the defect issue is generally critical in the ZnO:B-based devices, the carrier trapping characteristics in ZnO:B films is important. For thermal budget reduction, the as-deposited amorphous ZnO:B films grown by RF magnetron sputtering are concerned. In this work, the trap exploration in the amorphous boron-doped ZnO films was studied. An asymmetric metal-oxide-metal (MOM) structure with ZnO:B was fabricated and investigated for the studies of metal/oxide interface property and defect trap nature in ZnO:B films. The structure of Ni/ZnO:B/TaN was used. Based on the analysis of current-voltage (*I-V*) characteristics, Ohmic conduction is obtained at positive bias voltage due to the low work-function electrode TaN which forms Ohmic contact at the TaN/ZnO:B interface. Whereas, hopping conduction dominates at negative bias voltage due to the defective traps in ZnO:B in which the trap energy level is lower than the energy barrier at the Ni/ZnO:B interface. Lower energy obstacle leads to higher carrier transport, and therefore dominates the conduction current through the oxide. In the hopping conduction, the temperature dependent *I-V* characteristics reveal that the higher temperature, the lower current. This implies that the current decrease at higher temperature results from the multiple-level traps existed in the amorphous ZnO:B films.

## 2. Results and Discussion

**Figure 1:**
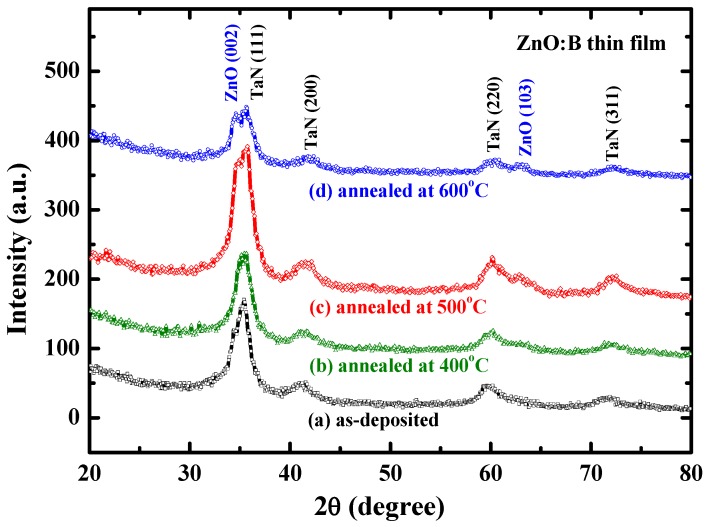
X-ray patterns of boron-doped ZnO films: (**a**) as-deposited; (**b**) annealed at 400 °C; (**c**) annealed at 500 °C; and (**d**) annealed at 600 °C.

To examine the microstructure of boron-doped ZnO (ZnO:B) films, the X-ray diffraction (XRD) patterns were measured at room temperature using a powder diffractometer (Cu target, 45 kV, 40 mA, scanning speed = 3°/min, scanning ranged from 2*θ* = 20° to 2*θ* = 80°, (PANalytical, Almelo, The Netherlands). According to the experimental results, carbon contamination level was extremely low in our films deposited from the ZnO:B source material and was below the detection limit of the Auger electron spectroscopy system (ULVAC-PHI , PHI 700, Kanagawa, Japan). Figure 1 depicts the indexed XRD spectra for as-deposited and annealed ZnO:B films. According to the XRD spectra, the intensity related to (002) and (103) planes for the as-deposited and 400 °C-annealed samples is too weak to deduce the polycrystalline phase in ZnO: B film. Thus, this figure reveals that the as-deposited ZnO:B film is amorphous and becomes polycrystalline when annealing temperature is above 500 °C. The polycrystalline ZnO:B film has a strong (002) peak and a weak (103) peak, similar to the results in a previous work [9]. These X-ray peaks result from the hexagonal wurtzite structure of ZnO with preferred orientation along *c*-axis. The mean grain size of both 500 °C- and 600 °C-annealed ZnO:B films estimated by Scherrer formula according to the full-width at half-maximum (FWHM) of (002) peak in Figure 1 is around 11 nm [9]. Herein, the trap investigation and conduction mechanism concentrated primarily on as-deposited amorphous ZnO:B films. Aside from the peaks of ZnO:B in Figure 1, the other four peaks located at 35.9°, 41.7°, 60.4° and 72.3° correspond to the (111), (200), (220) and (311) planes of the cubic structure of the TaN bottom electrode, respectively. The TaN electrode has a preferential orientation in the (111) direction.

**Figure 2:**
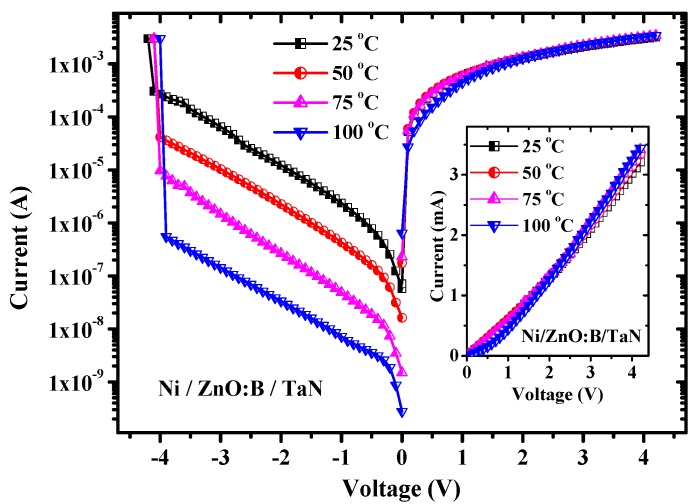
Temperature dependence of *I-V* characteristics in Ni/ZnO:B/TaN devices. The inset indicates the Ohmic behavior at positive bias voltage.

**Figure 3:**
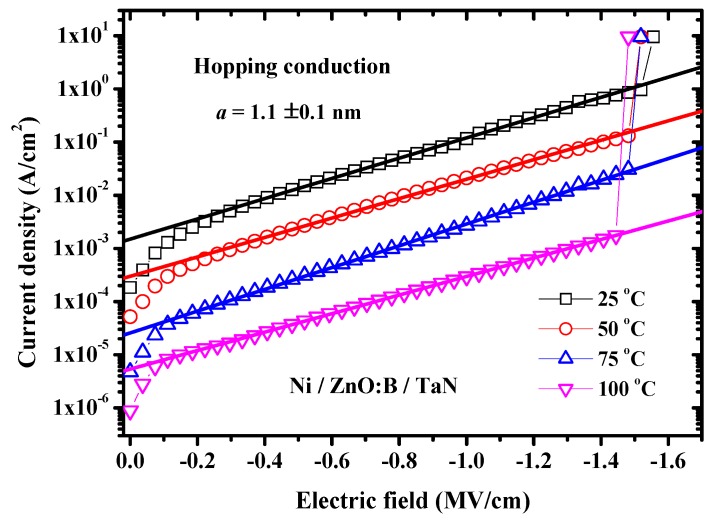
Temperature dependence of *J-E* characteristics in Ni/ZnO:B/TaN devices at negative bias voltage. The straight lines indicate the hopping conduction mechanism.

**Figure 4:**
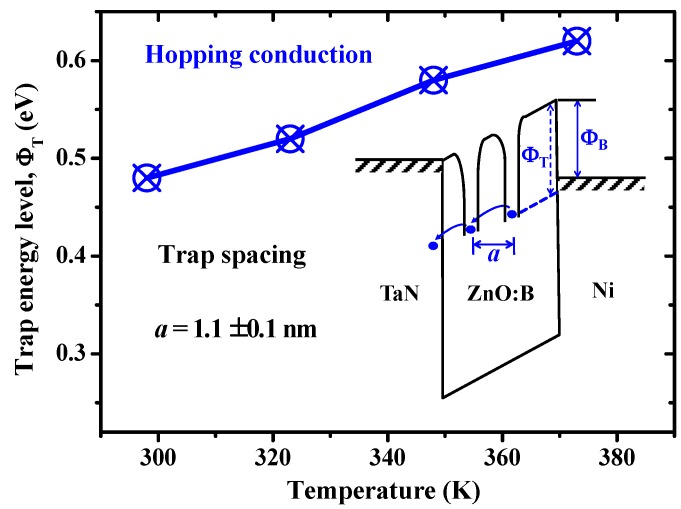
Temperature dependence of trap energy levels in boron-doped ZnO film. Inset shows the band diagram of hopping conduction in Ni/ZnO:B/TaN structure.

Since metal-oxide interface plays an important role in current conduction in a metal-oxide-metal structure, different metal electrodes are adopted to investigate the carrier transportation in this work. Nickel (Ni) is a high work-function metal and its value is 5.15 eV [11]. Whereas, tantalum nitride (TaN) is a low work-function electrode and its value is 4.15 eV [12]. Hence, the asymmetric structure of Ni/ZnO:B/TaN capacitors were fabricated in this work. Because ZnO:B is an n-type semiconductor and its electron affinity is 4.1–4.2 eV [13,14], Ohmic TaN/ZnO:B contact can be obtained due to low work-function of TaN. On the contrary, the Ni/ZnO:B interface yields an energy barrier due to high work-function of Ni [15]. Figure 2 shows the temperature dependence of *I-V* characteristics in Ni/ZnO:B/TaN capacitors. Under positive bias, linear *I-V* behaviors are observed, as indicated in the inset of Figure 2. The current conduction yields the Ohmic nature as a consequence of Ohmic contact at the TaN/ZnO:B interface. Meanwhile, under negative bias the non-linear *I-V* behaviors are obtained because of the electron energy barrier at the Ni/ZnO:B interface. In this case, there are a number of conduction mechanisms that may all contribute to the conduction current through the ZnO:B film at the same time. To distinguish these conduction mechanisms, measuring the temperature dependence on conduction current may afford us some valuable information to know the constitution of the conduction currents because several conduction mechanisms depend on the temperature in different ways [16]. Generally, a certain conduction mechanism may dominate the conduction current and the dominant conduction mechanism can be usually discovered after some typical analyses. In this work, the temperature dependence on *I-V* characteristics in Ni/ZnO:B/TaN structure is shown in Figure 2. According to the *I-V* characteristics at negative bias in Figure 2, the current level is lower as the temperature is higher. This nature is quite different from the normal *I-V* characteristics in oxide films in which the higher the temperature is, the larger the current has. Furthermore, the breakdown voltage of ZnO:B is around 4 V, namely, the breakdown field of ZnO:B is around 1.5 MV/cm. To investigate the current conduction mechanism in Ni/ZnO:B/TaN structure, oxide current simulations and typical plots of characteristic dependence on current density (*J*) and electric field (*E*) can be adopted [16]. The simulation results exhibit that the experimental data measured at negative bias match the theory of hopping conduction very well when the electric field is larger than about 0.2 MV/cm, as shown in Figure 3. Hence, the dominant conduction mechanism in Ni/ZnO:B/TaN structure at negative bias is the hopping conduction. The hopping conduction can be expressed as [16]:

(1) $$J=qanv\times \exp\left[\frac{qaE}{kT}-\frac{\Phi_{t}}{kT}\right]$$

where *q* is the electronic charge; *a* is the hopping distance (*i.e*., mean trap spacing); *n* is the electron concentration in the conduction band; *v* is the frequency of thermal vibration of electrons at trap sites; *E* is the applied electric field; *T* is the absolute temperature; *k* is the Boltzmann’s constant; and $\Phi_{t}$ is the energy level from the trap states to the bottom of the conduction band (*E_C_*). In this work, the electron concentration is about 10^18^ cm^−3^ in the ZnO:B films according to the Hall measurement. Based on Equation (1), the mean trap spacing can be determined by the slope of the linear part of log(*J*) *versus E* at each temperature. Hence, the trap spacing in ZnO:B films is extracted to be 1.1 ± 0.1 nm according to Figure 3. In hopping conduction, the electron energy is lower than the maximum energy of the potential barrier between two trapping sites, as shown in the inset of Figure 4. Therefore, the electron transport in ZnO:B films results from the tunneling effect in oxide films. Based on Equation (1), the hopping conduction current depends mainly on both the field energy ($\Phi_{E}$) induced from *qaE* and the trap energy level $\Phi_{t}$ in oxide films. If $\Phi_{E}$ > $\Phi_{t}$, then the hopping conduction current decreases with increasing temperature. On the contrary, the hopping conduction current increases with increasing temperature when $\Phi_{E}$ < $\Phi_{t}$. Taking the conditions of the largest electric field (*i.e*., breakdown field 1.5 MV/cm) and average hopping distance (1.1 nm), the maximum field energy $\Phi_{E\max}$ is around 0.165 eV. This indicates that the hopping conduction current will increases with increasing temperature when $\Phi_{t}$ is larger than 0.165 eV and the other parameters are fixed. However, the device current exponentially decreases with temperature in this work, as shown in Figure 3. Thus we consider that the trap energy level in ZnO:B films is not a constant but increases with temperature. Before the simulation work for determining the trap energy levels in ZnO:B films, the electron concentration and the frequency of thermal vibration of electrons at trap sites need to be resolved. In this work, the electron concentration is about 10^18^ cm^−3^ in the ZnO:B films according to the Hall measurement. Moreover, the frequency of thermal vibration of electrons at trap sites can be qualitatively represented by the frequency of optical phonons in the solid [17]. The phonon notion is generally associated with a super-lattice structure (polycrystalline materials). Although the range order in amorphous solids is smaller than that in polycrystalline ones, the phonon concept is also used in this work. The literature on the energy of optical phonon in ZnO is approximately within the range of 300–600 cm^−1^ [5]. Namely, the frequency of optical phonons in ZnO is around 1–2 × 10^13^ Hz. In this work, the frequency of 1 × 10^13^ Hz was assumed. The deviation induced from the uncertainty of optical phonon frequency in ZnO:B films is smaller than 0.02 eV. This value in the determination of trap energy level can be neglected in this work. Hence, the temperature dependence of trap energy levels in ZnO:B films were obtained, as shown in Figure 4. According to the simulation work, the trap energy level increases with temperature. This suggests that there are traps with deeper energy level incited by the elevated temperature. Accordingly, these incited traps lead to the exponential decrease in current at higher temperatures. This phenomenon is also observed in W/ZnO:B/W and Pt/MgO/Pt structures in which the resistive switching behavior was revealed [9,18]. In this work, the trap energy levels obtained in the simulation work are approximately within the range of 0.46 eV and 0.64 eV at 25–100 °C. These trap levels are close to the ones in defects of neutral zinc interstitials (Zn_i_), singly charged zinc interstitials (Zn_i_^+^), and doubly charged zinc vacancies (V_Zn_^2−^). Zn_i_, Zn_i_^+^, and V_Zn_^2−^ are all the defects of non-lattice ions in ZnO films. The defect energy levels of Zn_i_, Zn_i_^+^, and V_Zn_^2−^ in ZnO films are 0.46, 0.5, and 0.56 eV, respectively [19]. This result suggests that the defects of Zn_i_, Zn_i_^+^, and V_Zn_^2−^ may play the important roles in the current conduction in ZnO:B films. These defects may be introduced during the ZnO:B deposition process. Consequently, not single-level but multiple-level traps were found in the amorphous ZnO:B films. Note that defects, such as interstitials and vacancies, are imperfections in the crystal lattice. Interstitials signify extra atoms occupying interstices in the lattice. Meanwhile, vacancies signify missing atoms at regular lattice positions. In addition, current flow through the oxides will be raised at higher temperature for oxide films with the single-level traps. Based on our previous study [20], the single-level traps with 0.46 eV exist in the non-doped ZnO films in which the current density in a high resistance state increases with increasing temperature. However, Figure 3 shows that the current density decreases with increasing temperature in the boron-doped ZnO films due to the multiple-level traps. Because these two device fabrication processes are similar except the doping condition, we consider that the multiple-level traps come from the boron doping process in this work and are the origin of current reduction at higher temperatures in Ni/ZnO:B/TaN structure under negative voltage bias.

**Figure 5:**
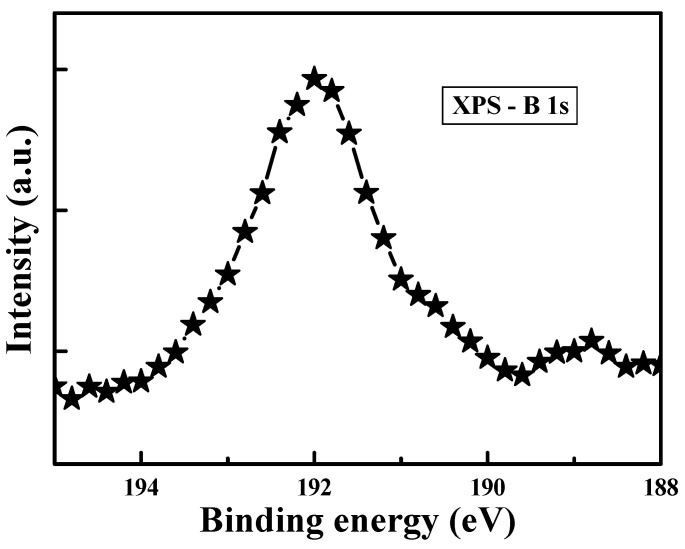
XPS spectrum of B 1s in boron-doped ZnO film deposited by RF magnetron sputtering.

**Figure 6:**
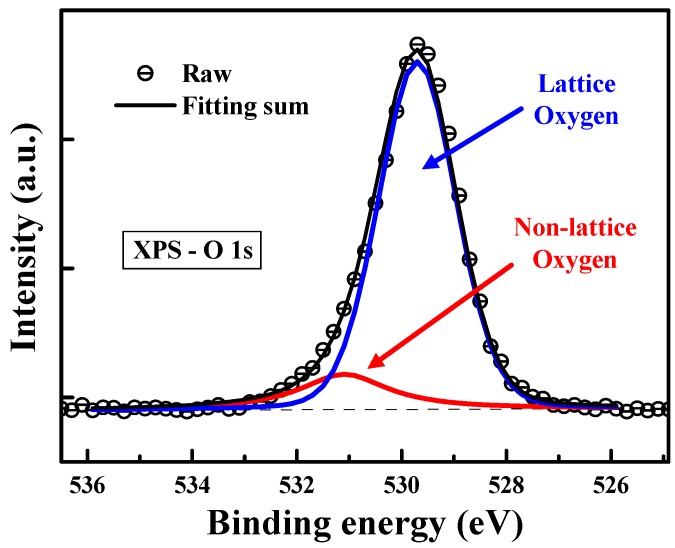
XPS spectrum of O 1s in boron-doped ZnO film deposited by RF magnetron sputtering.

**Figure 7:**
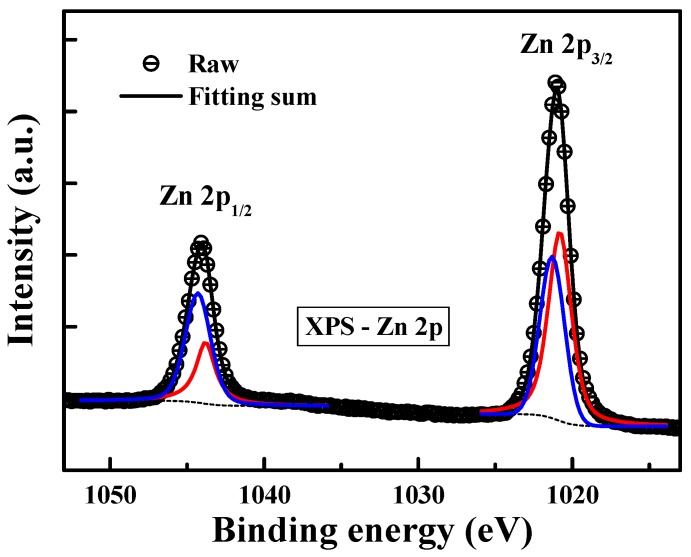
XPS spectrum of Zn 2p in boron-doped ZnO film deposited by RF magnetron sputtering.

According to the study of current conduction mechanisms in this work, we revealed that the trap energy levels in ZnO:B films are around in the range of 0.46–0.64 eV below the conduction band edge (*E*_C_). To explore the chemical defects in the amorphous ZnO:B films, the X-ray photoelectron spectroscopy (XPS) spectra were used to examine the chemical states of zinc and oxygen. Thermo Fisher Sceientific Theta Probe XPS system (with Al K_α_ source, Waltham, MA, USA) was used to collect the photoelectron spectra of the samples with a take-off angle of 90° relative to the sample surface. The vacuum pressure was below 10^−9^ torr during spectra data acquisition and using high resolution scans (0.02%~2%). In order to obtain meaningful binding energies, charge referencing was performed for the XPS measurements. In the beginning of the XPS measurements, the binding energy of the photoelectron was calibrated by assigning 284.8 eV to the C1s peak corresponding to adventitious carbon. For detecting the binding energies in the middle part of ZnO:B films, XPS spectra were collected after sputter-cleaning with 1-keV Ar^+^ ions for 1.2 min. Figure 5 shows the XPS spectrum of B 1s in ZnO:B film. The binding energy peak located at approximately 192 eV is associated with the B^3+^ in B_2_O_3_ structure, which provides the evidence for the incorporation of boron into the zinc oxide [21]. Figure 6 shows the O 1s XPS spectrum of the amorphous ZnO:B films. The profile of the O 1s spectrum was fitted using the Lorentzian–Gaussian functions. The binding energy peaks located at 529.7 [22] and 531.1 eV are attributed to lattice oxygen (ZnO) and nonlattice oxygen (oxygen vacancy) ions, respectively. Figure 7 shows the Zn 2p double spectra of the amorphous ZnO:B films. The binding energies of Zn 2p_1/2_ and 2p_3/2_ for Zn^2+^ correspond to the peaks at 1044.7 and 1021.5 eV, respectively [23,24]. Meanwhile, the peaks of binding energies of Zn 2p_1/2_ and 2p_3/2_ for nonlattice zinc ions are located at 1043.9 and 1021.1 eV, respectively. According to Figure 6 and Figure 7, the peak intensity (Zn 2p_1/2_ and 2p_3/2_) of nonlattice zinc ions is much more obvious than that (O 1s) of nonlattice oxygen ions. Namely, the peak area ratio of nonlattice to lattice zinc ions is much higher than that of nonlattice to lattice oxygen ions. This implies that the number of zinc deficient states is much larger than that of oxygen deficient states. As a consequence, the defects related to nonlattice zinc ions play the more important role in the current conduction in the amorphous ZnO:B films. A literature report pointed out some defect energy levels regarding nonlattice zinc and oxygen ions [19]. The defects related to nonlattice zinc ions include neutral zinc interstitials (Zn_i_), singly charged zinc interstitials (Zn_i_^+^), and doubly charged zinc vacancies (V_Zn_^2−^). The defects related to nonlattice oxygen ions include neutral oxygen vacancies (Vo), singly charged oxygen vacancies (Vo^+^), and oxygen interstitials (O_i_). The defect energy levels of Zn_i_, Zn_i_^+^, and V_Zn_^2−^ are 0.46, 0.5, and 0.56 eV, respectively. Furthermore, the defect energy levels of Vo, Vo^+^, and O_i_ are 1.62, 2, and 2.28 (or 2.96) eV, respectively. According to the simulation work on the hopping conduction current in ZnO:B films, the trapping level of the multiple-level traps is about in the range of 0.46–0.64 eV at 25–100 °C. This result also suggests that the defects related to the nonlattice zinc ions play the key role in the current conduction in the amorphous ZnO:B films. Note that the location of oxygen vacancy state can correspond to the activation energy obtained in the experiments. Aside from ZnO films in this work, the activation energy values in various dielectric materials can be found in the literature. One of the references indicated that the activation energy values of the different oxygen defects are in the range of 0.1–0.5 eV for the singly ionized oxygen vacancies, 0.6–1.2 eV for the doubly ionized oxygen vacancies, and 0.9–1.1 eV for the diffusion oxygen vacancies [25].

## 3. Experimental Section

In this work, the metal-oxide-metal (MOM) capacitors were fabricated. The boron-doped ZnO (ZnO:B) thin films were deposited on TaN/SiO_2_/Si substrates by radio frequency (RF) magnetron sputtering in argon ambient at room temperature using a ceramic ZnO:B target. The boron doping concentration of ZnO:B films was about 0.8 wt %. The flow rate of argon was 20 standard cubic centimeters per minute (sccm). The working pressure during deposition was 4 mTorr. The RF power was 60 W. The deposited ZnO:B film thickness was 27 nm. To investigate the crystal properties of ZnO:B films, rapid thermal annealing (RTA) was performed in N_2_ for 30 s at temperatures ranging from 400 °C to 600 °C. To achieve the MOM structure, a nickel (Ni) top electrode was deposited by thermal evaporation with a round area of 3.14 × 10^−4^ cm^2^ patterned by the metal shadow mask. The electrical characteristics of the fabricated Ni/ZnO:B/TaN capacitors were measured by an semiconductor parameter analyzer (Agilent 4156 C, Hachioji, Japan). During the measurement, the voltage bias was applied on the top electrode (Ni) with the bottom electrode (TaN) grounded. All the measurements were performed under dark condition.

## 4. Conclusions

In conclusion, the trap properties in amorphous boron-doped ZnO films were studied using the structure of Ni/ZnO:B/TaN. The Ohmic conduction dominates at positive bias voltage; meanwhile, the hopping conduction dominates at negative bias voltage. Based on the analysis of current conduction mechanism, we revealed that the defects with multiple trap levels exist in the amorphous ZnO:B films. These defects are related to the nonlattice zinc ions and play the key role in the current conduction. Simulation results show that the defect trap spacing between the defect sites is around 1.1 nm. Furthermore, the defect trap level increases with increasing temperature.
